# Supplementary material for: Sex and estrous cycle affect experience-dependent plasticity in mouse primary visual cortex
Source: PLoS One. 2023 Apr 17;18(4):e0282349. doi: 10.1371/journal.pone.0282349 (PMC10109517; doi:10.1371/journal.pone.0282349)
Supplement: Attachment — Submitted filename: Response to Reviewers_2.pdf [file pone.0282349.s001.pdf]

Responses to Journal Requirements:

1. Our revied manuscript carefully follows the style guide.
2. This work was funded by two NIH grants, one from NIMH and one from the NEI. Neither institute had a role in study design, etc. nor did they pay salary. Here is the funding statement:
   This work was funded by grants NIMH R00MH099654 and NEI R01EY030200. Neither institute took part in study design, data collection, or analysis or paid author salary.
3. Data not shown statements been removed from the paper. All data used for statistics, including both raw and normalized data, is available online via a link in the manuscript.
4. Our study did not use human subjects and needs no IRB approval or consent forms. The manuscript already includes details of our IACUC approved protocols.
5. Our reference list is correct.

Responses to Reviewer's Comments:

Reviewer 1: We appreciate viewer 1's enthusiastic support for this work.

Reviewer 2:

1. Good suggestion, figure 2 has been modified.
2. The statistical details are included in the text, but at the reviewer's suggestion we have also added indicators marking statistical difference to figures 3 and 4 and have also modified the figure legend to indicate significant differences.
3. Statistics, including n values, are reported in the text for all data. Following the reviewers comment we also added them to plot legends in figures 1, 3 and 4.
4. From the methods section: "Unless otherwise noted, 2-way ANOVAs were used to determine the statistical impact or either sex or estrus stage on stimulus evoked VEP potentiation as a function of either training day or stimulus type and the Shapiro-Wilk test was used to confirm data normality."
5. For simplicity, in cases where normalized and raw data lead to the same statistical conclusions figure showed only the normalized plots. The average relative magnitudes of raw voltage traces can be seen on the voltage plots and all data (raw and normalized) are available for download from the repository cited in the paper. At the reviewer's suggestion we added an explicit reference to figure 3C where significant day 5 differences are shown.
6. There is a small difference between male and female mice in sequence learning. The data shows that visual sequences drive slightly larger VEP responses in male populations than females, and that this difference accounts for the statistical effect found between the groups when calculated with raw (but not normalized) data. The details of this difference are included in the manuscript.
7. The difference between male and female mice found for raw voltages (see above) suggests sequence learning is more sensitive, but the 2-day induction experiment does not show increased sensitivity to estrous cycle. Overall the differences are small and can be controlled via normalization. The ratio of day 5 to day 1 was slightly larger in

males (2.47x vs 2.38x) but this was not a large enough difference to make a statistical difference with normalized data.

8. Generally, no. The physiological differences are small enough that any functional dimorphism would likely be essentially de minimis. Confirming this with functional behavioral assays is beyond the scope of this work.
9. VEP morphology changes in a characteristic manner with electrode depth. Electrodes were placed at the same depth relative to the cortical surface in all mice which was confirmed via postmortem histology.
